# White matter microdissection of the medial aspect of the brain: 2-dimensional video demonstration

**DOI:** 10.1007/s00701-026-06791-w

**Published:** 2026-03-03

**Authors:** Luca Zanuttini, Victor E. Staartjes, Grazia Menna, Shao-Ching Chen, Chun-Fu Lin, Sanford P. C. Hsu, Carolina Martins, Hung Tzu Wen, Giovanni Colacicco, Paulo A. S. Kadri, Niklaus Krayenbühl, Carlo Serra, Uğur Türe

**Affiliations:** 1 Machine Intelligence in Clinical Neuroscience & Microsurgical Neuroanatomy (MICN) Laboratory, Department of Neurosurgery, Clinical Neuroscience Center, University Hospital Zurich, University of Zurich, Frauenklinikstrasse 10, 8091 Zurich, Switzerland; 2 Department of Biomedical and Neuromotor Sciences (DIBINEM), University of Bologna, Bologna, Italy; 3 Department of Neurological Surgery, Fondazione Policlinico Universitario Agostino Gemelli, Rome, Italy; 4 Department of Surgery, Division of Neurosurgery, Cheng Hsin General Hospital, Taipei, Taiwan; 5 Division of General Neurosurgery, Neurological Institute, Taipei Veterans General Hospital, Taipei, Taiwan; 6 Neurology and Neurosurgery Unit, Hospital das Clínicas, Federal University of Pernambuco, Recife, Brazil; 7 Division of Neurosurgery, Hospital das Clínicas, College of Medicine, University of São Paulo, São Paulo, Brazil; 8 Institute of Anatomy, University of Zurich, Zurich, Switzerland; 9 Department of Neurosurgery, Mackenzie University Hospital, Curitiba, Brazil; 10 Department of Neurosurgery, University Children’s Hospital Zürich and University of Zürich, Zurich, Switzerland; 11 Department of Neurosurgery, Yeditepe University School of Medicine, Istanbul, Turkey; 12 Present Address: Department of Clinical Neuroscience, Karolinska Institutet, Stockholm, Sweden

**Keywords:** Klingler, White matter, Microneurosurgery, Fiber dissection, Limbic lobe

## Abstract

**Background:**

White matter microdissection represents a valuable method for studying the three-dimensional organization of the human brain. Building on Klingler’s classical technique, this approach has regained importance as both a research and educational tool in microneurosurgery. While the white matter dissection of the lateral aspect of the brain has been extensively described, the medial surface has received comparatively less attention. This article represents the second part of a three-paper series on white matter microdissection. It provides a structured, step-by-step demonstration of the dissection of the medial aspect of the brain, with a specific focus on the limbic lobe. As in the first paper, the core content is the supplementary video, which, through editing and annotation, has an enhanced didactic value.

**Methods:**

The dissection was performed according to the Klingler technique and recorded during the 8th *Sulci, Gyri, Ventricles and Fiber Dissection Hands-on Course* (Taipei, 2025). The procedure was conducted under microscopic magnification and edited into a didactic video presentation. All identifiable participants provided informed consent for publication.

**Results:**

The dissection proceeds through nine main stages, beginning with the identification of the medial sulci and gyri and continuing with the sequential removal of cortical layers and white matter bundles. This systematic approach exposes the cingulum, inferior longitudinal fasciculus, callosal fibers, hippocampal formation, tapetum, thalamic peduncles, and mammillothalamic tract, illustrating the organization of the limbic lobe and the relationships between major white matter tracts and the ventricular walls.

**Conclusion:**

This work presents a comprehensive and didactic video demonstration of the medial hemispheric white matter dissection, highlighting the continuity and spatial relationships of limbic and periventricular structures. It contributes to a systematic and anatomically grounded understanding of cerebral white matter organization and supports the educational role of white matter microdissection in neurosurgical anatomy.

**Supplementary Information:**

The online version contains supplementary material available at 10.1007/s00701-026-06791-w.

## Introduction

Over the past decades, the microdissection of cerebral white matter has become an established method for exploring the three-dimensional organization of the human brain grey and white matter structures [2, 6, 11–15, 27, 29, 30]. Building on Klingler’s classical preparation technique and its refinement under the operating microscope, this approach has regained importance as both research and an educational tool in modern microneurosurgery [1, 9, 20, 21, 32, 37].

The dissection of the medial aspect of the brain primarily focuses on the limbic lobe, highlighting the architecture of the hippocampal formation and its associated fiber systems. Understanding the spatial relationships between white and gray matter components within this region is essential to appreciate the structural complexity of the limbic network, which plays a pivotal role in memory, emotion, and behavior [10]. From a clinical perspective, detailed knowledge of the limbic lobe provides insight into the characteristic compartmentalization of intrinsic tumors, which often remain confined within its cytoarchitectonic boundaries during early stages of progression [3, 4, 38]. Moreover, a precise topographic understanding of medial hemispheric anatomy is fundamental for the microneurosurgical management of deep-seated lesions, allowing for safer surgical planning and preservation of surrounding structures [31, 33, 37].

Compared with the lateral surface [40], the medial dissection has been described far less frequently in the literature. Most anatomical studies have focused on isolated components of the limbic system rather than presenting an integrated view of its white matter organization [2, 11, 18, 29]. To our knowledge, no step-by-step video-based demonstration has previously been reported.

The present work addresses this gap by providing a structured, didactic demonstration of the medial hemispheric dissection, based on previous seminal works [32, 37]. Its main contribution lies in the supplementary video, edited and annotated to emphasize key anatomical and educational aspects. This article represents the second part of a planned three-paper series on white matter microdissection, following our previous report on the lateral aspect of the hemisphere [40].

## Materials and methods

The present white matter microdissection was recorded during the 8th *Sulci, Gyri, Ventricles and Fiber Dissection Hands-on Course* (Taipei, November 2025). All identifiable participants provided informed consent for publication of their images. The dissection refers to a single hemisphere prepared and dissected by the senior author (C.S.) and was performed under microscopic visualization with a Zeiss OPMI Pentero surgical microscope. Video editing and post-processing were carried out using Final Cut Pro (Apple Inc.), while schematic overlays were created using Microsoft PowerPoint to enhance didactic clarity.

The specimen was prepared according to the Klingler protocol, consisting of fixation, freezing and thawing, and dissection [9]. The details of this procedure have been described in our previous report on the lateral aspect of the hemisphere [40]. Summarily, the brain was fixed in 10% formalin for at least two months, frozen at –10 to –15 °C for a minimum of one week and thawed in water before dissection. Between sessions, the specimen was stored in 5% formalin.

Dissection was performed with wooden spatulas and suction under the operating microscope, with the adjunct of fine forceps and a scalpel when required. Continuous irrigation with water was used to preserve tissue hydration.

## Results

This section outlines the main steps of medial brain dissection, integrating technical tips derived from the Zurich laboratory’s experience. A detailed demonstration of each step is provided in Supplementary Video 1.

### Step 1 – Detailed study of sulcal and gyral anatomy

The dissection begins with a meticulous analysis of the medial sulcal and gyral anatomy. The cingulate pole and the medial paraolfactory gyrus are first identified between the posterior paraolfactory sulcus and the cingulate sulcus, being separated by the anterior paraolfactory sulcus. The paraterminal gyrus lies anterior to the medial paraolfactory gyrus. The rostral gyrus is located between the cingulate pole and the gyrus rectus, delimited by the superior and inferior rostral sulci. Posteriorly, the cingulate gyrus extends between the callosal and cingulate sulci. This structure represents a mesocortical component of the limbic lobe, interposed between the allocortical formations of the hippocampal region and the neocortex of the frontal, parietal, and occipital lobes.

The precuneus is located between the marginal ramus of the cingulate sulcus and the parieto-occipital fissure. The isthmus constitutes the narrow posterior end of the cingulate gyrus, lying just behind the splenium of the corpus callosum, and continues, together with the anterior portion of the lingual gyrus, into the parahippocampal gyrus. Finally, the fusiform gyrus is found between the collateral and the lateral temporo-occipital sulci.

The structures inferior to the cingulate sulcus and medial to the collateral sulcus, together with the precuneus, pertain to the limbic lobe, forming a continuous cortical arch from the subcallosal area to the parahippocampal region.

### Step 2 – Decortication of the medial aspect of the brain

After identifying the main sulcal and gyral landmarks described in the previous step, the decortication of the medial surface is performed. The cortex is removed with a wooden spatula or gentle suction, taking particular care due to the thinness of the limbic mesocortex compared with the lateral neocortex.

The U-fiber layer, composed of short association fibers connecting adjacent gyri, is correspondingly thinner on the medial surface. Decortication should therefore proceed slowly and with utmost care, avoiding excessive traction that may disrupt the underlying white matter.

### Step 3 – Removal of U-fibers and the cingulum to expose callosal fibers and ILF

After decortication, the U-fibers connecting the cingulate gyrus with the adjacent frontal and parietal cortices are removed. Entering the callosal sulcus with a wooden spatula, the longitudinal fibers of the cingulum are gently separated from the deeper layer of callosal fibers.

The cingulum consists of associative fibers running from the subcallosal gyrus to the amygdala, coursing along the depth of the cingulate and parahippocampal gyri. It can be divided into an anterior and a posterior portion, separated at the level of the cuneus apex. A sharp incision with a scalpel is made to detach the bundle in both directions, following its course.

Before proceeding with dissection, the parieto-occipital fissure is examined. This fissure is among the deepest in the brain and has been shown in anatomical studies to be consistently uninterrupted. It extends anteriorly to reach the calcarine fissure, dividing it into anterior and posterior segments.

Removal of the U-fibers on either side of the fusiform gyrus exposes the inferior longitudinal fasciculus (ILF). The thin central portion of this bundle allows its trajectory to be followed along the basal surface of the temporal and occipital lobes. The ILF represents a long associative system connecting non-adjacent gyri within these regions.

### Step 4 – Removal of the subiculum to expose the dentate gyrus

The collateral sulcus serves as a reliable landmark to identify the temporal horn from the basal surface of the brain. If necessary, the mesencephalon can be removed with a scalpel to facilitate visualization of the mesial temporal structures, particularly the retrocommissural hippocampal formation [11].

By gently pulling down the subiculum with a spatula inserted into the hippocampal sulcus, the entire structure of the retrocommissural hippocampal formation can be examined. The subiculum represents its outer extraventricular component, as demonstrated in the video. The other two extraventricular components are the margo denticulatus (corresponding to the dentate gyrus) and the fimbria, a white matter bundle composed of hippocampal efferent fibers that continues posteriorly as the crus of the fornix. The fimbriodentate sulcus lies between the margo denticulatus and the fimbria, while the choroidal fissure corresponds to the cleft between the fimbria–fornix complex and the thalamus.

The intraventricular component of the hippocampus, not yet visible at this stage, is composed of the CA1, CA2, and CA3 fields of the Ammon’s horn and the dentate gyrus. As illustrated in the video, CA1, CA3, and the dentate gyrus terminate anteriorly in the uncinate gyrus, the band of Giacomini, and the intralimbic gyrus, respectively. Posteriorly, they continue into the gyri of Anders Retzius, the fasciola cinerea, and the gyrus fasciolaris. These three posterior gyri are collectively referred to as the subsplenial gyrus, which forms the transition between the retrocommissural and supracommissural hippocampus.

The supracommissural hippocampal formation comprises the indusium griseum, a thin layer of grey matter overlying the callosal fibers, and two thin bundles of white matter known as the longitudinal striae or nerves of Lancisi, both clearly visible in the video demonstration.

The subiculum is carefully depressed along the hippocampal sulcus and eventually removed to expose the anterior portion of the medial temporal lobe. The uncus can be divided into two components. The anterior part harbors the amygdala and is composed of the semilunar gyrus and the ambiens gyrus, separated by the semiannular sulcus. The posterior part contains the head of the hippocampus within the tip of the temporal horn and is formed by the uncinate gyrus, the band of Giacomini, and the intralimbic gyrus.

### Step 5 – Removal of the retrocommissural hippocampal formation to expose the ventricle

The hippocampal formation is detached from the tip of the temporal horn and peeled away together with part of the crus of the fornix, demonstrating the close anatomical relationship between these two structures. A transverse incision is made through the head of the hippocampus to display its internal organization.

### Step 6 – Removal of the supracommissural hippocampus to expose callosal fibers

The supracommissural hippocampus, composed of the indusium griseum and the longitudinal striae, is gently peeled away from the underlying callosal fibers. The transverse orientation of these fibers becomes clearly evident at this stage.

### Step 7 – Removal of the corpus callosum and the ependyma to expose the caudate nucleus and fibers on the lateral ventricular wall

The corpus callosum is then removed with a scalpel in a piecemeal fashion, completing the unroofing of the frontal horn, cella media (body), and atrium of the lateral ventricle and exposing its entire lateral wall.

The choroid plexus and the fornix are detached from the choroidal fissure by releasing the teniae fornicis and choroidea.

From this perspective, the 4 “free thalamic surfaces” [30] and their boundaries are visible. Noteworthy structures include the stria terminalis and the superior thalamostriate vein, running in the striothalamic sulcus covered by ependyma, between the caudate nucleus and the lateral ventricle surface of the thalamus; the choroidal fissure, between the latter surface and the velar surface of the thalamus; the stria medullaris thalami, composed by fibers running between the septal region and the habenula, which separates the third ventricle and the velar surface of the thalamus. The velar surface of the thalamus corresponds to the lateral wall of the velum interpositum; postero-inferior to it is located the cisternal surface.

On the roof of the temporal horn, two longitudinal structures can be identified. Medially, the stria terminalis runs toward and terminates in the dorsomedial aspect of the amygdala, where it joins the central and medial amygdaloid nuclei, forming the so-called extended amygdala. Laterally, the tail of the caudate nucleus can be followed to its termination just anterior to the tip of the temporal horn.

The ependyma is removed from the ventricular wall, exposing the caudate nucleus and the tapetum, which embraces the lateral wall of the atrium and the temporal horn. The tapetum, from the Latin *tapetum*, meaning “carpet”, is formed by splenial fibers directed toward the temporal lobe.

Deeper and vertically oriented fibers are visible on the wall of the body and frontal horn of the lateral ventricle. These belong to the corona radiata, a system of projection fibers connecting the cerebral cortex with diencephalic structures above the internal capsule, which remains covered by the caudate nucleus at this stage.

### Step 8 – Removal of caudate and tapetum to expose the thalamic peduncles

The caudate nucleus is removed gently with a spatula to fully expose the corona radiata, which is composed medially by the fibers of the thalamic peduncles. The tapetum is also removed. The thalamic peduncles are thalamocortical and corticothalamic projections divided in 4 capsular components: the anterior, superior, posterior, and inferior thalamic peduncles. The posterior thalamic peduncle includes a loop on the roof and lateral wall of the lateral ventricle, well-known as the Meyer’s loop and relevant because it harbors the optic radiation, between other fibers of the sublenticular portion of the internal capsule.

### Step 9 – Exposure of the extracapsular thalamic peduncle and the mammillothalamic tract

Removal of the grey matter of the medial thalamic nucleus from the wall of the third ventricle exposes the fibers of the extracapsular thalamic peduncle, which connects the thalamus with other limbic and deep nuclei while bypassing the internal capsule. Among these fibers are the amygdalothalamic fibers, coursing infero-laterally posterior to the column of the fornix and eventually looping around the cerebral peduncle, together with the amygdaloseptal fibers arising from the septum verum. Collectively, these amygdalofugal fibers form the ansa peduncularis, linking the amygdala to distinct deep grey matter structures [19, 29].

The final step consists of removing the fibers of the extracapsular thalamic peduncle, thereby exposing the mammillothalamic tract (bundle of Vicq d’Azyr), which connects the mammillary body to the anterior thalamic nucleus.

## Discussion

This study provides a step-by-step demonstration of the white matter microdissection of the medial aspect of the brain, with a specific focus on the anatomy of the limbic lobe. By combining fiber microdissection with a didactic video presentation, we aimed to illustrate the three-dimensional organization of white and gray matter in this region and to offer an educational tool for both trainees and practicing neurosurgeons. The medial surface, though less frequently dissected than the lateral aspect, represents a key area of interest because of its structural complexity and its central role in neurocognitive and pathological processes.

### The concept and anatomical framework of the limbic lobe

Although emphasized by seminal anatomists and later championed by neurosurgeons such as Yaşargil, the term “*limbic lobe*” has not been routinely adopted in neurosurgical nomenclature, where traditional lobar divisions remain predominant [38]. This limited use, however, risks underestimating the fact that the limbic lobe represents a distinct anatomical and, in certain respects, functional entity with specific surgical relevance.

The concept of the limbic lobe has evolved considerably since its first description by Broca, who, based solely on topographical observations, identified a cortical ring surrounding the corpus callosum and brainstem, forming the border (*limbus*, in Latin) of the cerebral cortex, the *grand lobe limbique *[7]. Subsequent developments, from the emotion-centered theories of Papez and MacLean [23, 26] to the integrative models proposed by Nauta and Nieuwenhuys [24, 25], progressively broadened the concept of the limbic system to include subcortical nuclei and brainstem connections. Nevertheless, the anatomical notion of a limbic lobe—as a distinct cortical domain—has remained a valid and conceptually robust framework.

In its current understanding, the limbic lobe comprises all non-isocortical cortices, including the olfactory and hippocampal allocortices and the transitional “mesocortical” regions (periolfactory, perihippocampal, and periallocortical areas such as the retrosplenial, presubicular, parasubicular, and entorhinal cortices), together with the basolateral amygdaloid components [16]. These territories share a unifying cytoarchitectural and myeloarchitectural continuity, as demonstrated by successive studies employing axonal degeneration, immunohistochemical, and molecular methods. This framework supports the view of the limbic lobe as a morphologically and developmentally coherent cortical complex bridging the allocortex and neocortex.

### Surgical and pathological relevance

From a neurosurgical perspective, the limbic lobe represents a fundamental anatomical and functional unit, though working in cortico-subcortical reentrants circuits together with other diencephalic structures. Yaşargil emphasized the importance of this concept as a guide for surgical strategy, given its link with the principle of pathoclysis, introduced by Vogt: “*certain physiochemical properties of nerve cells that share common morphological characteristics, and often constitute cytoarchitectonically definable areas, confer upon them specific susceptibilities to a variety of pathogenic agents*” [35, 38]. In other words, selective vulnerability of certain cortical types underlies the predilection of intrinsic pathologies, such as gliomas or epileptogenic processes, to involve limbic territories [8, 22, 34, 39].

While it is common thought that tumor spread follows white matter tracts, an alternative interpretation, supported by cytoarchitectural principles and recent studies, proposes that the pattern of progression reflects the cortical compartment of origin [28]. In this view, the underlying architecture of allo-, meso-, or neocortex determines the subsequent growth and compartmentalization of the lesion, at least in its initial phases [3, 4]. A clear three-dimensional understanding of the medial surface and its limbic organization thus provides valuable insight into tumor behavior, improving surgical planning and resection strategies.

Similarly, in epilepsy surgery, knowledge of the limbic lobe, defining a neuronal network, is essential for the anatomo-electro-clinical definition and understanding of the epileptogenic zone and the subsequent seizure propagation [5, 41].

### Educational value

Despite its clinical relevance, the detailed anatomy of the temporomesial and limbic regions remains inconsistently represented in contemporary literature, with substantial discrepancies in terminology and subdivision. Few modern works have provided a clear depiction of their inner organization [11, 17, 36], which complexity fascinated Klingler himself, who dedicated to them his first white matter dissection atlas “*Die makroskopische Anatomie der Ammonsformation*”, published in 1948 [18]. Interestingly, his pioneering dissection work led to collaborations with key figures in temporal lobe epilepsy such as Pierre Gloor, giving the neuroanatomical basis to subsequent electrophysiological studies [1, 19].

This work continues the approach introduced in our previous study on the lateral hemispheric dissection, combining static and dynamic representation of white matter anatomy [40]. The video demonstration allows a progressive and spatial understanding of the dissection steps, highlighting the relationships between cortical and subcortical structures and their position with regard to the ventricular system. The integration of step-by-step fiber dissection and video narration facilitates comprehension of spatial organization in a way that static illustrations alone cannot achieve.

### Limitations

This study, as any cadaveric dissection, presents intrinsic limitations. The quality of anatomical detail depends on tissue preservation and fixation, and interindividual variability may influence the morphology of some structures. Moreover, the absence of functional correlation prevents any inference on connectivity or physiological relevance. However, as for all white matter dissections, the aim of this work is not to define function, but to provide a precise understanding of the topographical relationships among fiber bundles. Within these limits, stepwise dissection remains the most effective method to appreciate the spatial organization of the medial aspect of the hemisphere and its anatomical complexity.

## Conclusion

This work provides a detailed, stepwise demonstration of the white matter microdissection of the medial aspect of the brain, with particular emphasis on the anatomy of the limbic lobe. The integration of microscopic dissection and video documentation offers an accessible educational framework for appreciating the spatial organization of cortical, subcortical, and ventricular structures in this region.

The medial dissection, less frequently described than the lateral one, highlights the continuity and topographic relationships among the main components of the limbic lobe, a cortical domain bridging allocortical and neocortical territories and playing a central role in both physiological and pathological processes. This perspective allows a clearer understanding of the medial hemispheric anatomy and its internal organization, which are often difficult to appreciate through conventional anatomical descriptions or imaging-based reconstructions.

Within the limits of cadaveric anatomy, this study emphasizes the didactic and methodological value of white matter microdissection as a mean to explore the three-dimensional arrangement of the human brain. As the second installment of our series on white matter microdissection, it aims to promote a systematic and anatomically grounded approach to microneurosurgical education.

## Supplementary Information

Below is the link to the electronic supplementary material.

Supplementary file1 (MP4 1904957 KB)

## Data Availability

No datasets were generated or analysed during the current study.
